# Hierarchically structured 3D carbon nanotube electrodes for electrocatalytic applications

**DOI:** 10.3762/bjnano.10.146

**Published:** 2019-07-24

**Authors:** Pei Wang, Katarzyna Kulp, Michael Bron

**Affiliations:** 1 Martin-Luther-University Halle-Wittenberg, Faculty of Natural Sciences II, Department of Chemistry, 06120 Halle, Germany

**Keywords:** chemical vapor deposition, CNTs, CO stripping, hierarchically structured electrodes, methanol oxidation, platinum, poisoning tolerance

## Abstract

Hierarchically structured 3-dimensional electrodes based on branched carbon nanotubes (CNTs) are prepared on a glassy carbon (GC) substrate in a sequence of electrodeposition and chemical vapor deposition (CVD) steps as follows: Primary CNTs are grown over electrodeposited iron by CVD followed by a second Fe deposition and finally the CVD growth of secondary CNTs. The prepared 3-dimensional CNT structures (CNT/CNT/GC) exhibit enhanced double-layer capacitance and thus larger surface area compared to CNT/GC. Pt electrodeposition onto both types of electrodes yields a uniform and homogeneous Pt nanoparticle distribution. Each preparation step is followed by scanning electron microscopy, while the CNTs were additionally characterized by Raman spectroscopy. In this way it is demonstrated that by varying the parameters during the electrodeposition and CVD steps, a tuning of the structural parameters of the hierarchical electrodes is possible. The suitability of the hierarchical electrodes for electrocatalytic applications is demonstrated using the methanol electro-oxidation as a test reaction. The Pt mass specific activity towards methanol oxidation of Pt-CNT/CNT/GC is approximately 2.5 times higher than that of Pt-CNT/GC, and the hierarchical electrode exhibits a more negative onset potential. Both structures demonstrate an exceptionally high poisoning tolerance.

## Introduction

Carbon nanotubes (CNTs) have attracted considerable attention since their discovery in 1991 [1] due to their high electrical conductivity, large surface area, good chemical stability, high mechanical strength and high aspect ratio and are considered as promising materials for diverse applications such as field emission displays, energy storage devices, sensors, and so on [2–8]. Besides the above-mentioned applications, CNTs have also been investigated as catalysts or catalyst supports for various electrocatalytic reactions [8–13], including methanol oxidation in direct methanol fuel cells (DMFCs). DMFCs are promising power sources for future energy conversion and storage, since they, in addition to their nonpolluting nature and low operating temperature, run on an easily handled and cheap liquid fuel.

However, the slow kinetics of methanol oxidation at the anode and the methanol crossover through the electrolyte membrane from anode to cathode are still major obstacles that hinder the broad market implementation of DMFCs. The slow kinetics are mainly caused by incomplete methanol oxidation accompanied by the formation of adsorbed carbonaceous reaction intermediates, which poison the Pt surface [14–19]. Most strategies to solve these issues are focused on the optimization of the catalyst, such as alloying Pt with a second metal such as Ni, Ru and Pd [20–24] or using Pt-metal oxide composites such as Pt/SnO_2_ and Pt/CeO_2_ [24–28]. Additionally, a variety of catalyst preparation methods, e.g., colloidal synthesis [29–31], a galvanic replacement process [32–35] or microwave-assisted preparation [20–21,36], have been proposed to gain control over the structural features of the active nanoparticles.

However, from heterogeneous catalysis it is generally known that a suitable catalyst support is as important as the active material in order to form an optimum catalyst. For electrocatalytic applications, the support should possess high electrical conductivity, large surface area and good chemical and mechanical stability. Furthermore, the electrode prepared with the catalyst should provide optimized pore structure and retain the high surface area of the catalyst to guarantee a high availability of active sites and unhindered mass transport for high efficiency.

Besides the classical carbon blacks, different carbon-based catalyst supports (e.g., modified CNTs, functionalized reduced graphene oxide, etc.) have been recently studied to improve the reaction performance, enhance stability and thus reduce the cost [37–41]. It was reported that Pt supported on these optimized catalyst supports provides higher electrocatalytical activity towards methanol oxidation and increased tolerance against poisoning in comparison to those supported on carbon blacks and nonmodified catalyst supports. This could be due to improved Pt dispersion owing to a higher amount of functional anchoring sites of the catalyst supports and their high surface area, as well as from a good electrical contact between the conducting components [42–45]. The modification of the electronic and structural properties of Pt due to interaction with the support may also play a role.

To take advantage of the properties of novel carbon materials, and at the same time gain control over the electrode structure, bottom-up synthesis approaches have been suggested, including branching or hierarchical structuring of carbon-based catalyst supports. In these approaches, one-dimensional (1D, e.g., CNTs or nano-/microfibers) or two-dimensional carbon materials (2D, e.g., graphene) are transformed into three-dimensional (3D) structures by attaching other nanofibers or carbon materials. Examples are nanofibers distributed on polymer-based microfibers, CNTs grown on graphene, CNT–carbon black hybrids, graphene- or polymer-coated CNTs, and so on [45–51]. Another approach for hierarchical structuring is the growth of secondary CNTs on primary CNTs [52–56]. It was shown that such nanostructured CNT–CNT composites exhibit enhanced specific surface area as well as increased specific double-layer capacitance. Additionally, the presence of the secondary CNTs can reduce the equivalent series resistance to promote electron transfer. CNT–CNT composites have been successfully employed as catalyst supports. Kundu et al. reported that Pt supported on such hierarchical structures showed enhanced surface atomic concentration, indicating an improved Pt dispersion. The oxygen reduction reaction on Pt/CNT–CNT yielded a much higher diffusion-limited current compared to Pt supported on other carbon-based electrodes [52]. In general, CNT-based hierarchically nanostructured materials can be considered as promising support materials for electrocatalytic applications.

This paper investigates the preparation of hierarchically structured CNTs on glassy carbon (GC) based on a sequential CNT growth over electrodeposited Fe nanoparticles via chemical vapor deposition (CVD) with cyclohexane as the carbon precursor. Pt electrodeposition onto these hierarchical structures leads to active electrocatalysts. The bottom-up synthesis of these nanocomposites was monitored using scanning electron microscopy (SEM) and Raman spectroscopy, and it is demonstrated that the hierarchical structures can be tuned with respect to thickness, length, and density of the CNTs. The activity of the Pt-CNT/CNT/GC electrodes towards methanol oxidation was investigated and compared to that of Pt-CNT/GC and high activity and exceptional poisoning stability were demonstrated.

## Results and Discussion

### Preparation and characterization of hierarchically nanostructured electrodes

#### Fe deposition

**Figure 1:**
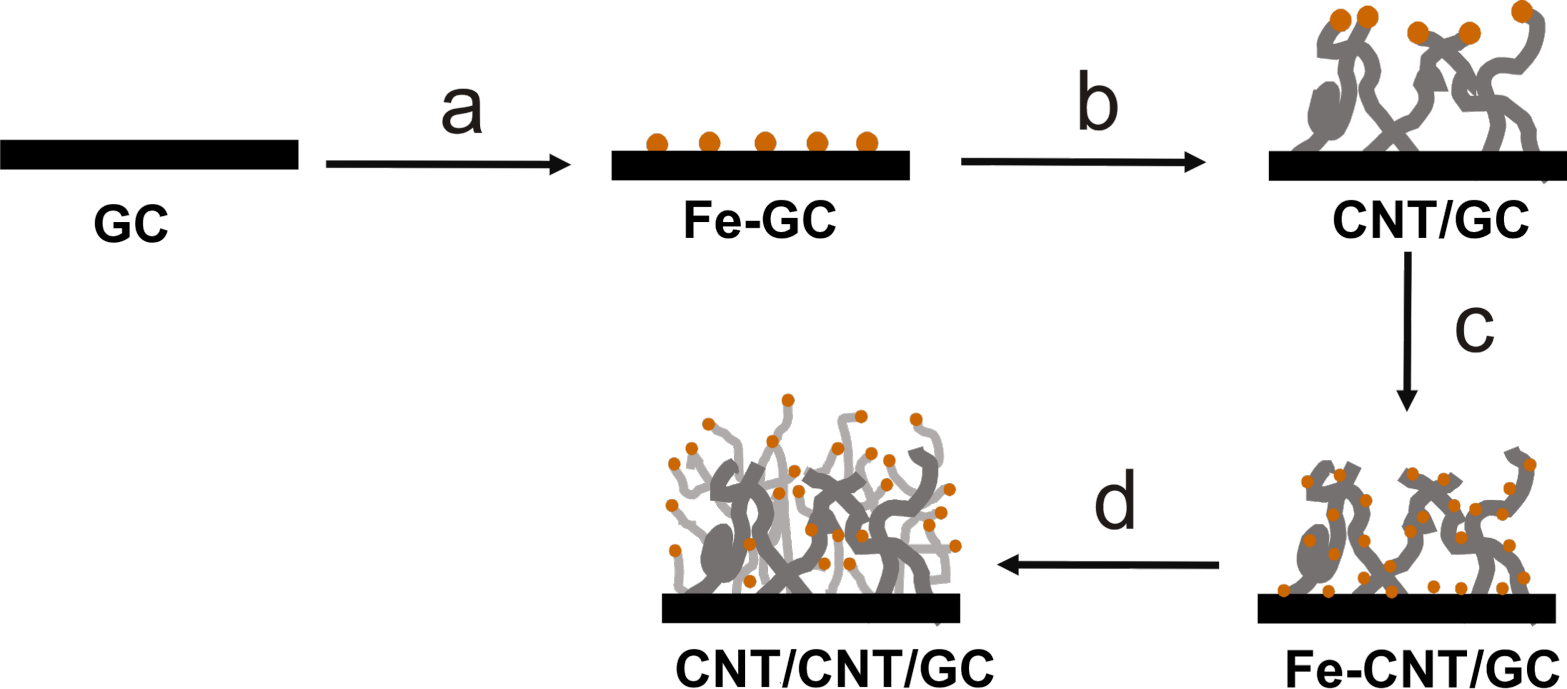
Scheme of the preparation of hierarchically nanostructured electrodes: a) electrochemical deposition of Fe nanoparticles onto oxidized GC; b) CNT growth onto GC through CVD; c) deposition of Fe nanoparticles onto CNTs and GC; and d) growth of secondary CNTs onto primary CNTs.

**Figure 2:**
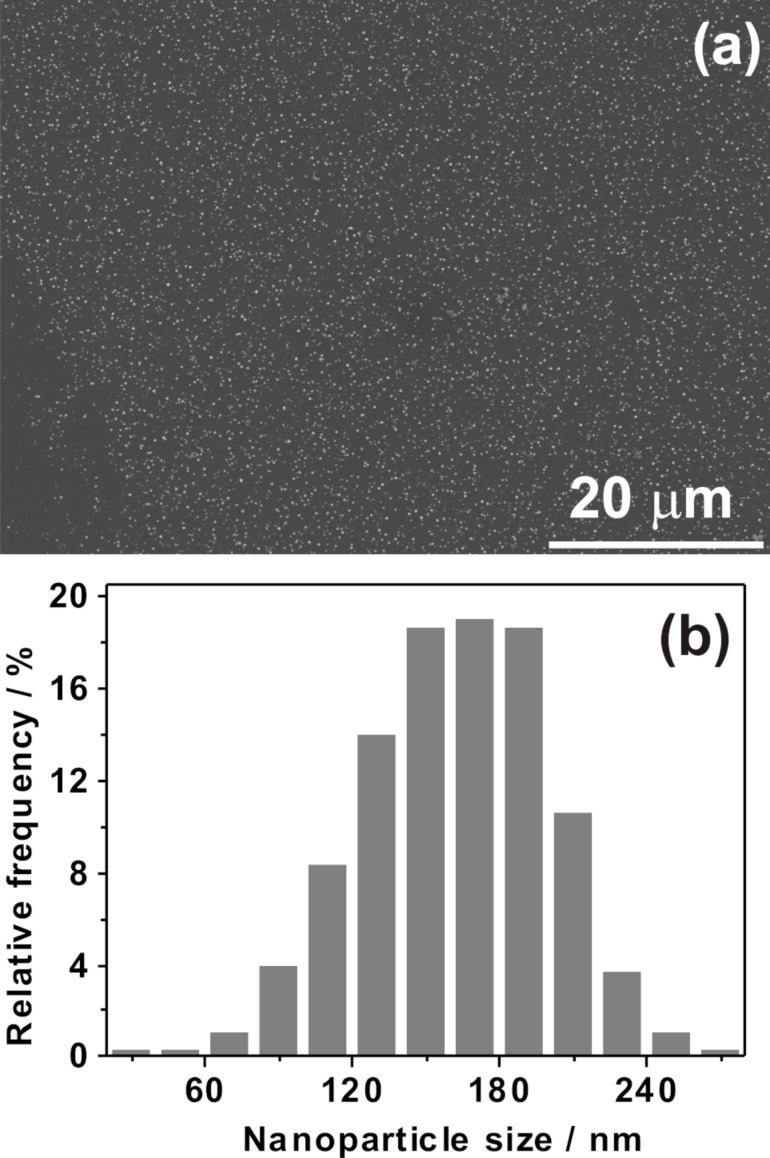
(a) SEM image and (b) particle size distribution of Fe nanoparticles electrochemically deposited onto GC.

In Figure 1, the individual steps for the preparation of hierarchically nanostructured electrodes are displayed schematically. First, Fe nanoparticles are electrodeposited onto oxidized GC followed by CVD growth of primary CNTs to form CNT/GC. After a second deposition of Fe nanoparticles, another CVD step leads to the hierarchically structured electrodes (CNT/CNT/GC). Each step has been optimized towards structural control and high reproducibility, as detailed below. The first and critical step is the initial Fe deposition. Fe nanoparticles were electrochemically deposited onto the GC surface using double pulse deposition [57]. This method allows adjustment of nucleation and growth potential to control the distribution and size of the Fe nanoparticles. A nucleation potential of −1.41 V vs Ag|AgCl|KCl_sat._ and a growth potential of −1.27 V vs Ag|AgCl|KCl_sat._ were applied (compare also Figure S1, Supporting Information File 1). Figure 2a shows an SEM image of Fe nanoparticles deposited onto oxidized GC. The particle diameter is in the range from 100–200 nm (Figure 2b), which is considerably large. Recent investigations in our lab, which will be published in the near future, show that particle sizes down to 20 nm and below are possible. Fe deposition onto nonoxidized GC is possible as well but leads to poor reproducibility and inhomogeneous samples with respect to particle size and size distribution. The control over the size of the particles is necessary since it was shown that the diameter of CVD-prepared CNTs can be associated with the size of the catalyst particles [58–59].

#### Growth of primary CNTs

After the metal catalyst deposition, the CNTs are grown via CVD. During CVD growth, the CNT structure (quality) and yield of CNTs is controlled by many parameters, such as the pressure, temperature, growth time, reactor geometry, carbon precursors, gas flow rate and composition of gas mixtures, as well as the catalyst support and physical and chemical state of the catalyst [59–63]. It is not the aim of this paper to present a detailed study on the influence of all these parameters. However, some of them turned out to be critical for the success or failure of the preparation of hierarchically structured electrodes, as detailed in the following.

**Figure 3:**
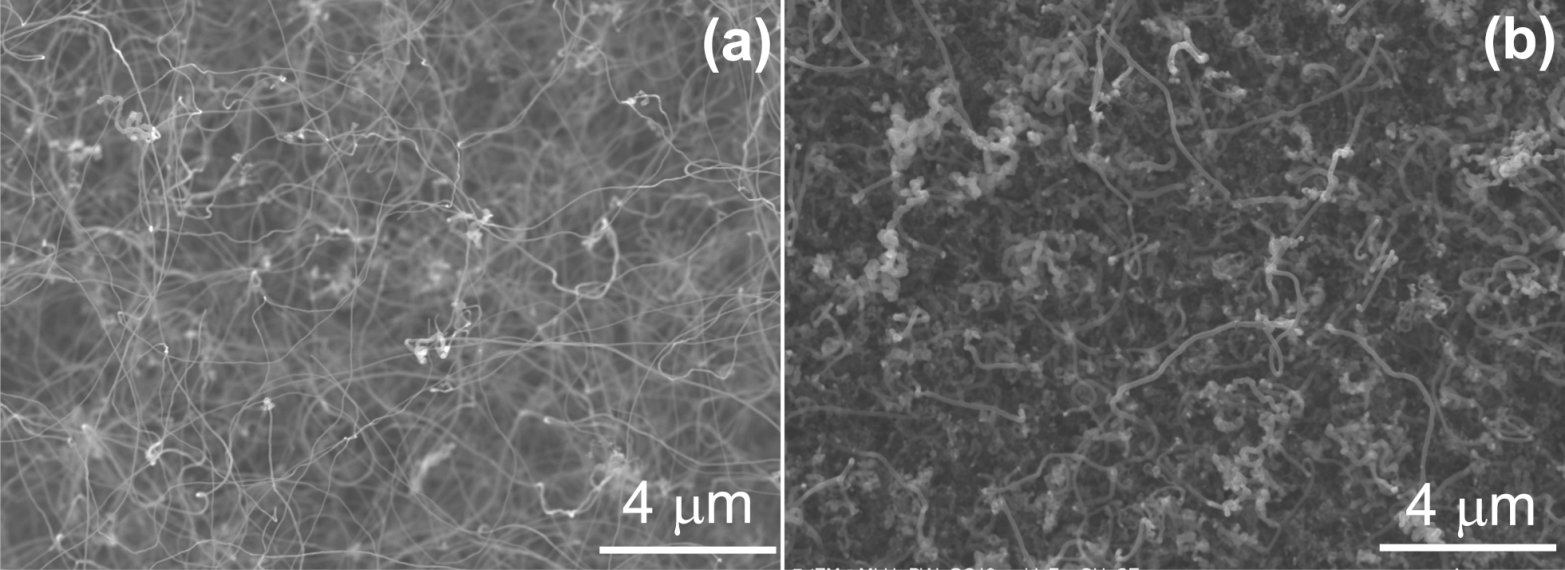
SEM images of CNTs deposited onto GC by CVD at 750 °C using cyclohexane and a gas flow rate of 1.7 L h^−1^ for 120 min with an H_2_/Ar ratio of 1.8 (1.1 L h^−1^/0.6 L h^−1^). (b) CNTs grown under the same conditions but with an increased total gas flow rate of 3.9 L h^−1^.

The CVD growth of primary CNTs over electrodeposited Fe nanoparticles was carried out with cyclohexane at 750 °C, a temperature that turned out to be suitable in reference experiments (not shown). Cyclohexane is brought into the CVD furnace using a H_2_/Ar gas mixture saturated at room temperature. To gain control over the CVD process, the influence of growth time, gas flow rate and H_2_/Ar ratio were studied. In a series of experiments, using a growth time of 120 min and a gas flow rate of 1.7 L h^−1^, the H_2_/Ar ratio is varied (which translates into a varied H_2_/cyclohexane ratio), and the results are represented in Figure 3a and Supporting Information File 1, Figure S2e and f. Using a H_2_/Ar ratio of 1.1 L h^−1^/0.6 L h^−1^ (Figure 3a), the primary CNTs were densely and nearly uniformly grown on the surface of GC with a diameter of approximately 40–80 nm. Accordingly, the optical image (Supporting Information File 1, Figure S2g) displays a matt black thin layer at those areas of the GC chips that were covered with Fe particles. However, no CNT growth was observed with a H_2_/Ar ratio higher than 1.2 L h^−1^/0.5 L h^−1^ (and thus a higher H_2_/cyclohexane ratio, Supporting Information File 1, Figure S2f), while Figure S2e shows only few CNTs and large amounts of surrounding (probably amorphous) carbon obtained with a smaller H_2_/Ar ratio (1.0 L h^−1^/0.7 L h^−1^). It was reported that the density and diameter of CNTs synthesized on carbon cloth with ethylene as the carbon precursor over a nickel catalyst at 700 °C decrease with decreasing ratio of H_2_ to N_2_ [62], while CNTs grown on an Fe-decorated Si wafer at 825 °C using toluene increased in density and diameter with decreasing ratio of H_2_/Ar [63]. This demonstrates that the CNT growth strongly depends on the growth conditions. Accordingly, the above-described results reveal the sensitivity of the CNT growth on the H_2_/cyclohexane ratio under the chosen conditions. During CVD growth, hydrogen molecules or atoms keep the metal catalyst in its active state and avoid catalyst passivation by excess carbon deposition, which would otherwise suppress CNT growth. We assume that with the decreasing ratio of H_2_/cyclohexane, exactly these processes occur, resulting in suppressed CNT growth and formation of amorphous carbon. In contrast, there is no CNT growth with the increasing ratio of H_2_/Ar, likely because excess hydrogen hydrogenates carbon structures formed at the catalyst surface into volatile compounds, thus hindering CNT growth.

The time dependence of CNT growth was examined for growth times ranging from 30 to 120 min (Figure 3a, Supporting Information File 1, Figure S2a,b). After 30 min of growth, no CNTs can be found, while short CNTs with a diameter of 40–80 nm are formed during 60 min of CVD growth. The diameter is similar to that of CNTs grown for 120 min (Figure 3a). From 30 min to 120 min, the density of the CNTs is increased. Obviously there is a considerably long conditioning period, during which the growth catalyst is likely slowly saturated with carbon until the optimum H_2_/cyclohexane (or carbon) ratio is reached.

Besides varying the H_2_/Ar ratio and the growth time, the influence of the total gas flow rate and thus the cyclohexane feed on CNT growth was studied using the optimum H_2_/Ar ratio (1.8) and a growth time of 120 min. Figure 3b shows CNTs grown with a total gas flow rate of 3.9 L h^−1^. The CNTs grow densely and homogenously with a diameter of approximately 150 nm. This is about twice as thick as the diameter of the CNTs grown with 1.7 L h^−1^. Most likely, the larger amount of decomposed carbon crystallizing on the Fe nanoparticles to form a cylindrical network is the reason for this observation. Using 6.7 L h^−1^and 12.1 L h^−1^ as the total gas flow rate, only few CNTs were grown (Supporting Information File 1, Figure S2c,d). We assume that the excess carbon surrounds the Fe nanoparticles, blocking them from further CNT growth. It might be speculated that a higher H_2_/cyclohexane ratio would allow CNT growth also at higher total gas flow rates, which we have not yet investigated. Regardless, the above results demonstrate that by choosing the appropriate experimental conditions, it is possible to tune the thickness and length of the primary CNTs grown on glassy carbon.

#### Growth of secondary CNTs and Pt deposition

**Figure 4:**
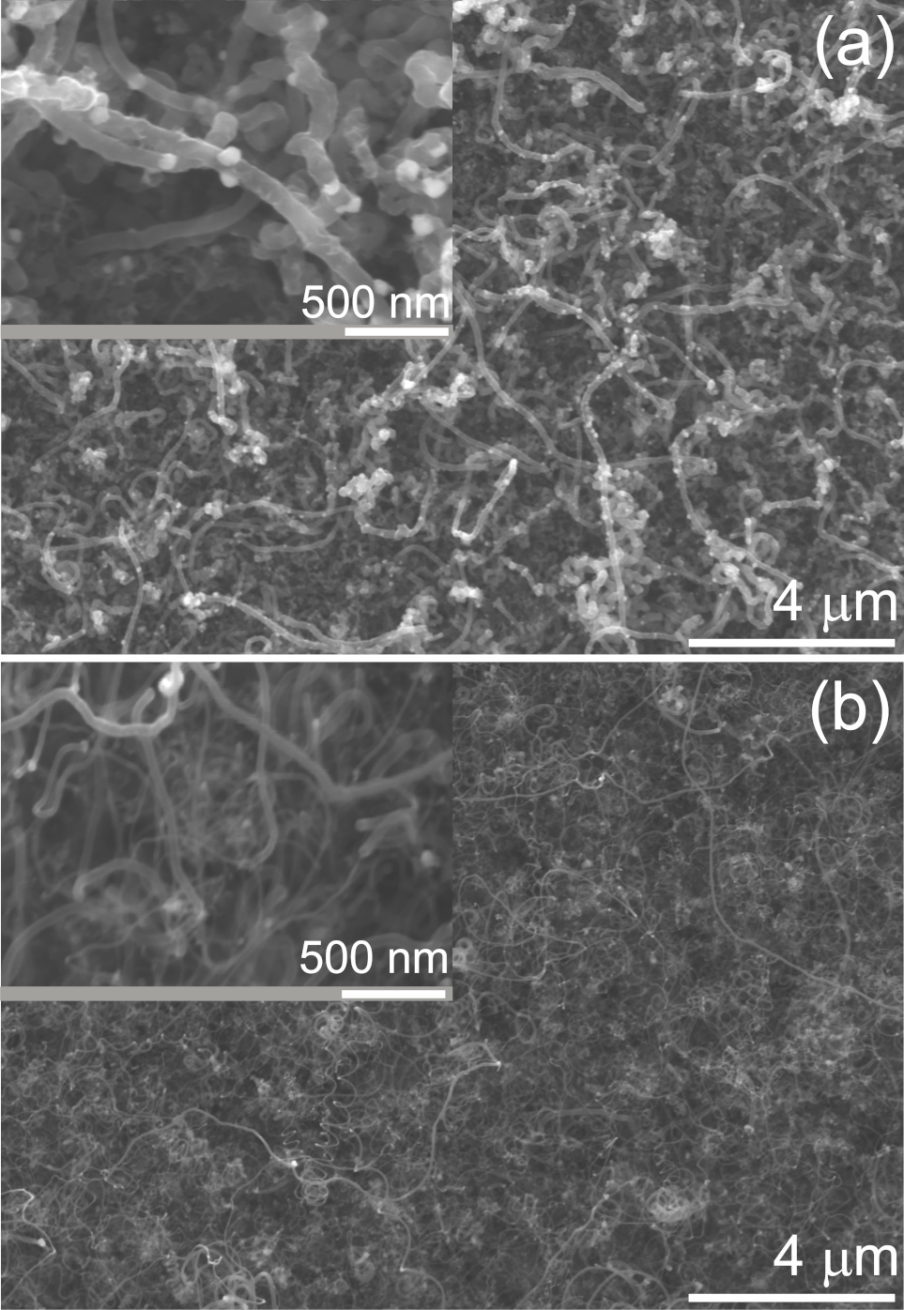
SEM images of (a) Fe nanoparticles electrodeposited onto primary CNTs and GC (8 s of deposition time) and (b) secondary CNTs grown at 750 °C for 120 min with a H_2_/Ar ratio of 2.4 (1.2 L h^−1^/0.5 L h^−1^).

After the growth of the primary CNTs, a subsequent Fe electrodeposition and growth of secondary CNTs was carried out to form the hierarchical electrodes (CNT/CNT/GC) as shown in the SEM images of Figure 4. These experiments were carried out with the thicker primary CNTs grown at a gas flow of 3.9 L h^−1^. Figure 4a shows Fe nanoparticles deposited onto the primary CNTs with quite homogenous distribution. Double pulse deposition, as described above, was utilized but the deposition time was decreased from 12 s to 8 s, resulting in a reduced Fe particle size range from 50–90 nm. The use of thicker CNTs as the primary material and smaller Fe particles for the secondary CNTs was chosen to obtain truly hierarchical structures, facilitating the verification of the growth of secondary CNTs. The particle size can be controlled via the deposition time, as shown in Supporting Information File 1, Figure S3, with average particle sizes of ≈45 nm after 6 s of deposition time and ≈110 nm after 12 s of deposition. Additionally, the Fe nanoparticles seem to prefer to nucleate on cross junctions between primary CNTs, as observed from Figure S3a, which could be caused by improvement of electron transfer or preferential nucleation sites.

The growth of secondary CNTs using the same optimized gas mixture as above and a gas flow rate of 1.7 L h^−1^ yielded unsatisfactory results. As exposed in Supporting Information File 1, Figure S4, larger amounts of amorphous carbon are deposited and only few CNTs are grown, indicating the dependence of CNT growth on support and structure. Learning from the results on the growth of primary CNTs, the H_2_/Ar ratio was adjusted to 1.2 L h^−1^/0.5 L h^−1^ to avoid formation of amorphous carbon, and the growth of secondary CNTs was successfully achieved, as demonstrated in Figure 4b. The secondary CNTs were grown quite irregularly, which may be caused by the size distribution of the Fe nanoparticles but also by the fact that the gas composition within the 3-dimensional structure of the primary CNTs may change due to cyclohexane consumption by the CVD process. However, the presence of a large number of thinner CNTs compared to the initial structures verifies the growth of secondary CNTs (compare also Supporting Information File 1, Figure S5). It is, however, considerably difficult to identify junctions between the primary and secondary CNTs, probably due to top growth and the high density of CNTs.

Furthermore, to access the generality of our approach, the above designed procedure was successfully employed to prepare nitrogen-doped nanostructured electrodes (N-CNT/N-CNT/GC) using acetonitrile (CH_3_CN) as the carbon precursors and nitrogen source instead of cyclohexane (see Supporting Information File 1, Figure S6).

#### Physicochemical characterization

**Figure 5:**
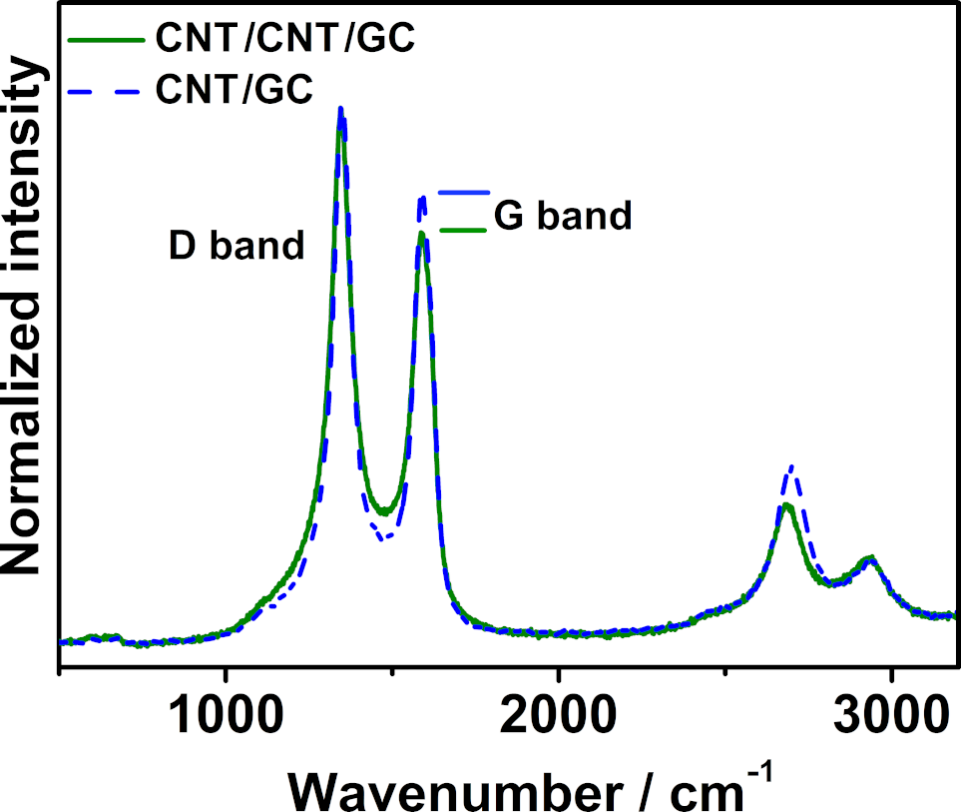
Raman spectra of CNT/GC and CNT/CNT/GC electrodes. The spectra are normalized with respect to the intensity of the D-band, and horizontal lines indicate the height of the G-band.

The prepared electrodes (CNT/GC and CNT/CNT/GC) were characterized by Raman spectroscopy (Figure 5) after Fe removal in concentrated HNO_3_ (before Pt electrodeposition). Both electrodes show the typical D-band at ≈1355 cm^−1^ und the G-band at ≈1600 cm^−1^, which are associated with structural defects within the carbon lattice and crystalline carbon, respectively [64]. The intensity ratios of these bands (*I*_D_/*I*_G_) for the CNT/GC und CNT/CNT/GC electrodes are 1.36 und 1.54, respectively. This indicates that the secondary CNTs are less ordered and have a higher defect density than the primary ones.

**Figure 6:**
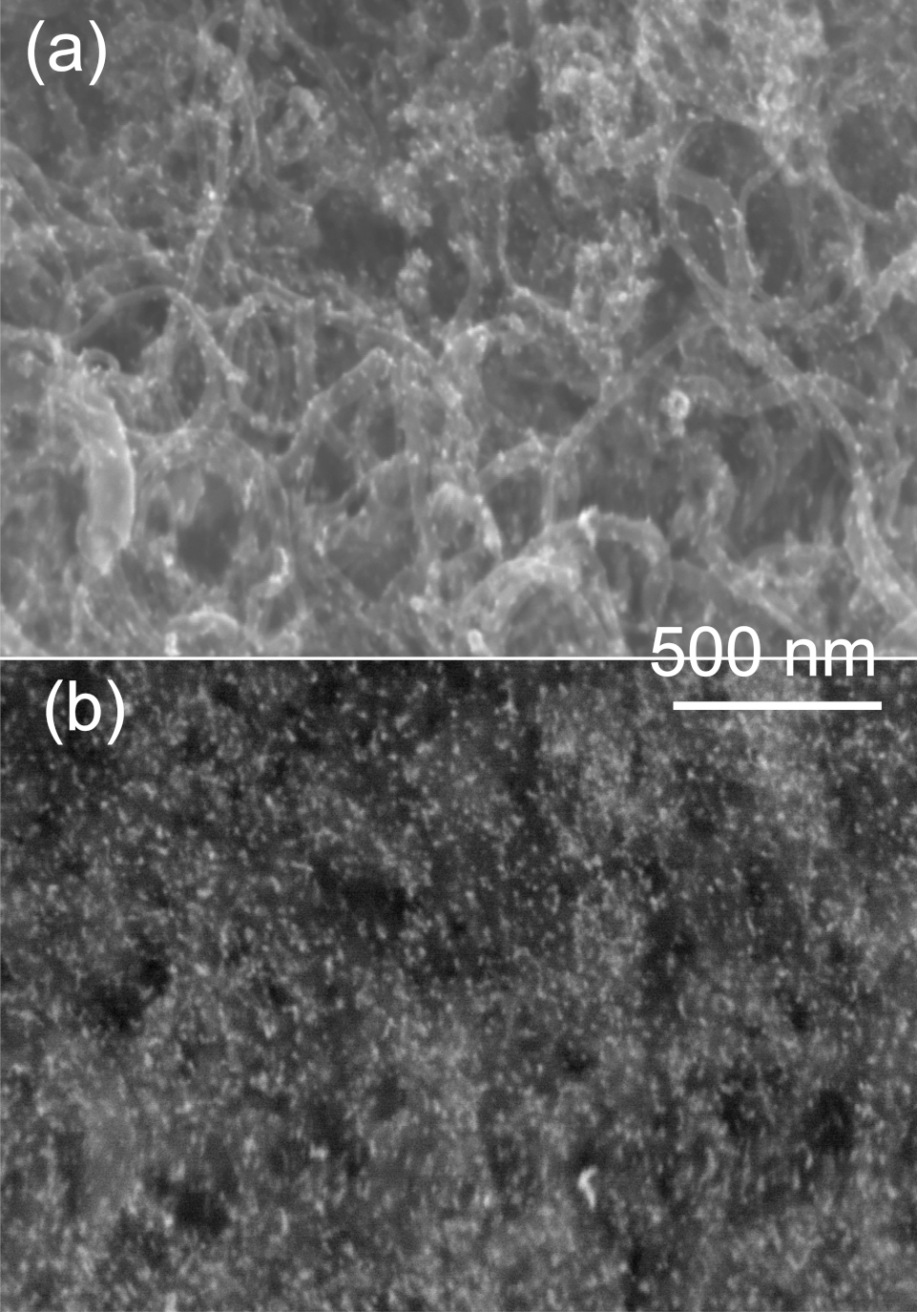
SEM (a) und BSE (b) image of Pt nanoparticles deposited from an aqueous 0.005 M Pt(NO_3_)_2_ and 0.1 M NaNO_3_ solution via single-sweep voltammetry from 0 to −0.9 V vs Ag|AgCl|KCl_sat._ at a scan rate of 5 mV s^−1^ onto CNT/CNT/GC.

As the last step in electrode preparation, Pt nanoparticles were electrochemically deposited onto CNT/CNT/GC and CNT/GC using linear-sweep voltammetry from 0 to −0.9 V vs Ag|AgCl|KCl_sat._. For comparison, Pt deposition onto oxidized GC was carried out in the same manner (compare Supporting Information File 1, Figure S7 for the resulting deposition curves). The very different double-layer capacities above −0.15 V are due to the different surface areas and was subtracted for charge integration. Based on Faraday’s Law and the charge consumed during the sweep, the mass of electrodeposited Pt onto the GC, CNT/GC and CNT/CNT/GC electrodes was calculated to be 0.147 mg, 0.101 mg and 0.065 mg, respectively (Table 1). It seems to be surprising that the amount of deposited Pt is highest on the sample with the lowest surface area. The reason for the decreasing Pt amount in the order GC, CNT/GC and CNT/CNT/GC is not clear to us at the moment; however, this was observed in repeated experiments. Similarly it was reported by Rajesh et al. [65] that the amount of electrodeposited Pt on graphene/CNT/GC was less than that on graphene/GC under the same deposition conditions. As shown in the SEM/BSE images in Figure 6 and Supporting Information File 1, Figure S8 and S9, Pt nanoparticles were homogenously and densely distributed onto the CNT/GC and CNT/CNT/GC electrodes with similar particle sizes (≈7 nm). Meanwhile, the Pt nanoparticles deposited on oxidized GC are much larger (≈50 nm, Supporting Information File 1, Figure S9). Besides electrodeposition onto CNTs, it may be assumed that some Pt is directly deposited onto the GC substrate. Furthermore, it cannot be excluded that particles smaller than the mentioned 7 nm form, which are below the detection limit of our SEM.

**Table 1:** Pt mass on the different supports and corresponding electrochemically active surface area (ECSA) determined by H_upd_ and CO_ads_.

	Pt-GC	Pt-CNT/GC	Pt-CNT/CNT/GC

Mass of Pt (mg) via LSV	0.147	0.101	0.065
ECSA from H_upd_ (cm^2^/mg)	1.11	6.81	12.39
ECSA from CO (cm^2^/mg)	–	10.95	13.87
Ratio of ECSA From CO vs H_upd_	–	1.61	1.12

In addition to SEM, XRD measurement were perfomed to analyze the Pt nanoparticles, however, no meaningful diffractograms were obtained due to the low overall Pt loading and probably due to the fact that the electrodeposited Pt nanoparticles seem to be quite irregular and might consist of several crystallites that are too small to be detected by XRD (compare Supporting Information File 1, Figure S8).

### Electrochemical Investigations

#### Cyclic voltammetry

**Figure 7:**
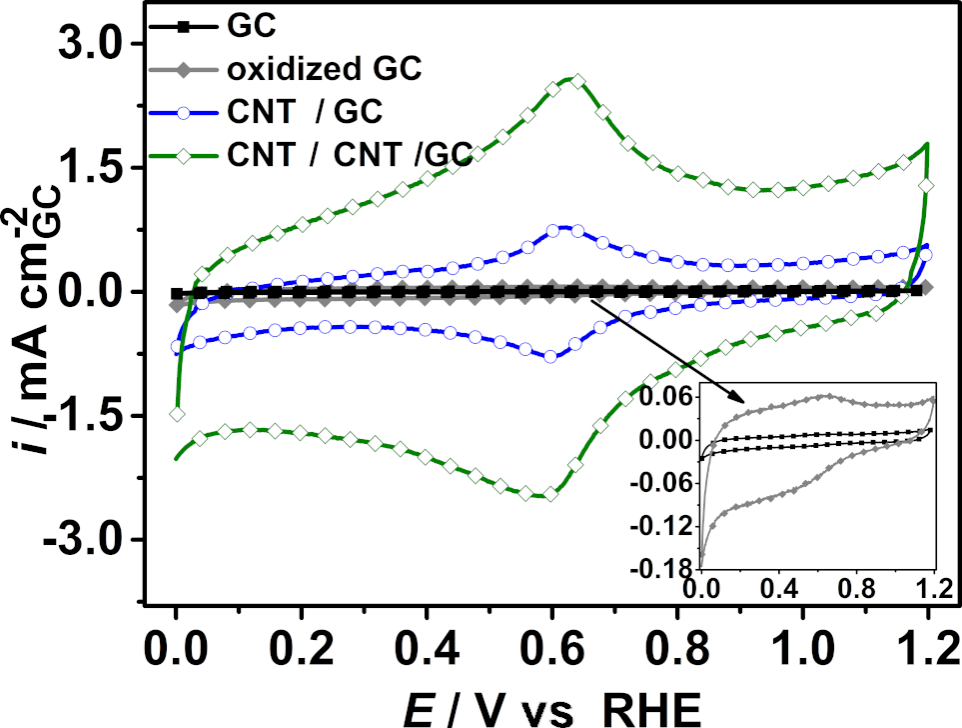
Cyclic voltammograms of GC, oxidized GC, CNT/GC and CNT/CNT/GC recorded at a scan rate of 100 mV s^−1^ in N_2_-saturated 0.5 M H_2_SO_4_ aqueous electrolyte solution at room temperature.

A basic electrochemical characterization of the prepared electrodes was carried out using cyclic voltammetry (CV). CVs of GC before and after oxidation in HNO_3_ as well as after growth of the primary CNTs and additional secondary CNTs are displayed in Figure 7. The currents in the CVs are associated with the charging and decharging of the electrical double layer and denote the double-layer capacity, which can be regarded as an estimation of the surface area for the carbon-only samples. In addition to these currents, in the potential range between 0.5 V and 0.7 V vs RHE, a redox peak pair is observed for all three samples, which is attributed to the presence of oxygen-containing groups (quinone-type) resulting from the necessary treatment with HNO_3_ (see experimental, preparation of GC surface or leaching of Fe particles) [66]. The double-layer capacity of oxidized GC is increased compared to GC before oxidation, which may be attributed to a roughening of the surface and probably the formation of oxygen-containing surface groups like –OH or –C=O [66]. After the CVD growth of the primary CNTs and the secondary CNTs, the double-layer capacity is significantly enhanced, demonstrating the successful CNT growth and the concomitant increase in the electrochemically available surface area. Additionally, the functional groups of the primary and secondary CNTs, which are formed in the concentrated HNO_3_ during the removal of Fe nanoparticles, can also contribute to the increase in the double-layer capacity [67]. The same observation can be made on the above-mentioned nitrogen-doped hierarchically nanostructured electrodes, where N-CNT/N-CNT/GC electrodes have a higher double-layer capacity (Supporting Information File 1, Figure S10). It should be mentioned that N-CNT/N-CNT/GC displays no redox peak attributed to oxygen-containing functional groups since Fe was electrochemically leached out in H_2_SO_4_ and not chemically in concentrated HNO_3_ for the sake of follow-up studies not presented here. Additionally, CNT/CNT/GC is hydrophobic, while N-CNT/N-CNT/GC turned out to be hydrophilic, rendering an oxidative treatment unnecessary.

**Figure 8:**
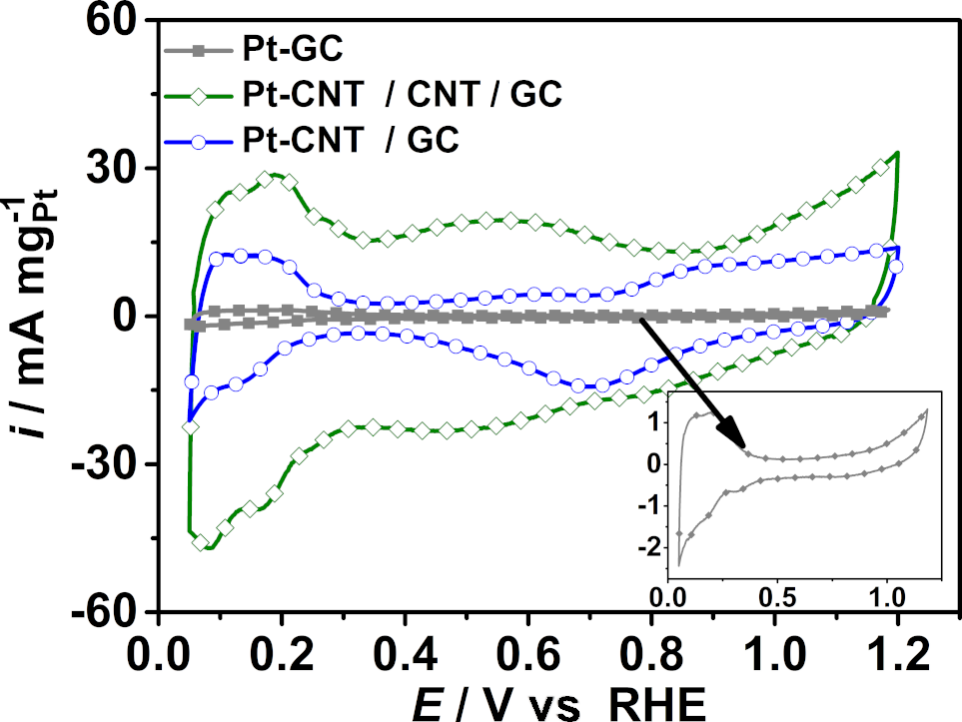
Cyclic voltammograms of Pt on GC, CNT/GC and CNT/CNT/GC electrodes recorded at a scan rate of 100 mV s^−1^ at room temperature in a N_2_-purged aqueous 0.5 M H_2_SO_4_ electrolyte solution.

As described above, Pt nanoparticles were electrodeposited onto the hierarchical electrodes and, for comparison, also onto the surfaces of oxidized GC and CNT/GC. The available catalyst surface area (electrochemically active surface area (ECSA)) of Pt on GC, CNT/GC and CNT/CNT/GC was determined from the H_upd_ charge and CO_ad_ stripping voltammograms. The respective cyclic voltammograms of H_upd_ were recorded in N_2_-saturated aqueous 0.5 M H_2_SO_4_ in the potential range from 0.05 to 1.2 V vs RHE at a scan rate of 100 mV s^−1^ as displayed in Figure 8. The ECSA was calculated from the average coulombic charge obtained via integrating the area under the hydrogen adsorption/desorption peaks after subtracting the double-layer charge in the potential range between 0.05 V and 0.35 V vs RHE [68–69]. As shown in Figure 8, the current density (normalized to Pt mass) of Pt-CNT/CNT/GC for hydrogen adsorption/desorption increases compared to Pt-GC and Pt-CNT/GC, and the determined values are displayed in Table 1. The increase in the ECSA for Pt-CNT/CNT/GC may be explained by the higher CNT surface area of CNT/CNT/GC as compared to CNT/GC, as deduced from the double-layer current. The secondary CNTs provide a larger number of anchoring sites (e.g., surface functional groups or junction between primary CNTs and secondary CNTs) to form a larger numbers of Pt nuclei during electrodeposition. As a consequence, the Pt particles in Pt-CNT/CNT/GC must be smaller than in Pt-CNT/GC. This difference is scarcely observed from the SEM images (Figure 6), which may be attributed to the limited resolution of SEM and non-observable Pt nanoparticles on GC. As described in the literature [52], secondary CNTs exhibit decreased charge transfer resistance with respect to the primary CNTs as determined by electrochemical impedance spectroscopy. Thus, we speculate that the improvement in Pt dispersion is due to a better conductivity within the 3D network and a facilitated electron transfer, which may facilitate Pt nucleation at the CNT surface. As expected, Pt-GC has a much lower ECSA compared to Pt-CNT/GC or Pt-CNT/CNT/GC, which is associated with the larger Pt nanoparticles (see Supporting Information File 1, Figure S9) resulting from the much lower surface area of GC.

#### CO stripping voltammograms

**Figure 9:**
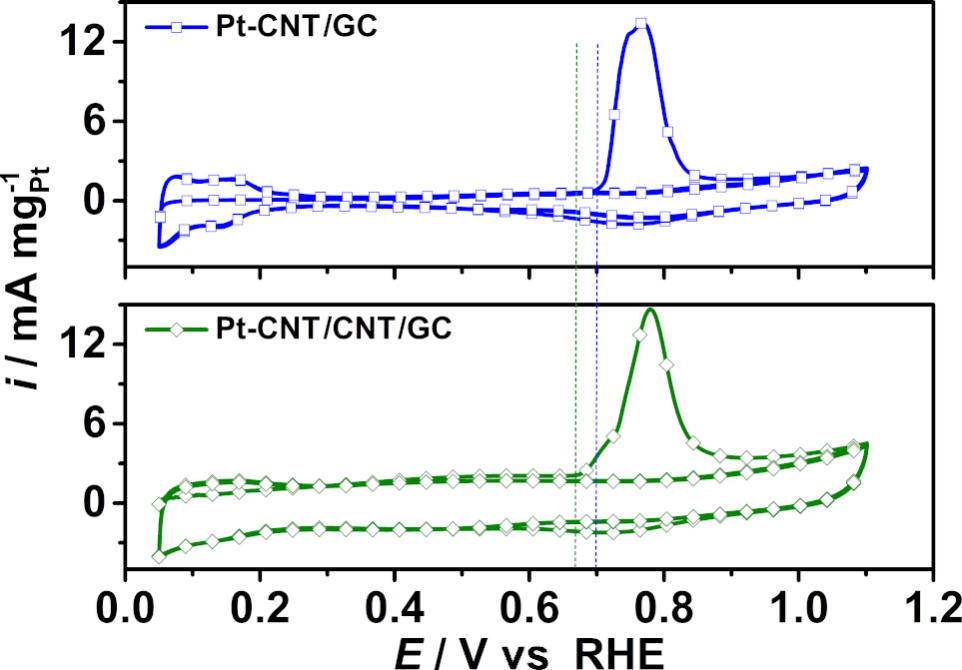
CO_ad_ stripping voltammograms of Pt-CNT/GC and Pt-CNT/CNT/GC monitored at 20 mV s^−1^ in CO-purged and subsequently N_2_-purged 0.1 M HClO_4_ solution. The vertical dashed lines are intended as a guide for the eye.

In addition, CO_ad_ stripping voltammograms were recorded at a scan rate of 20 mV s^−1^ in the potential range of 0.05–1.1 V vs RHE after CO adsorption in N_2_-purged 0.1 M HClO_4_ solution for ECSA determination as well as investigation of CO tolerance as shown in Figure 9. HClO_4_ was used as the electrolyte for these investigations instead of H_2_SO_4_ for a better comparability with literature values and to avoid disturbance of the CO_ad_ stripping voltamogramms by sulfate/bisulfate adsorption. The charge consumed during CO_ad_ oxidation was used to calculate the ECSA, and the values are 10.95 cm^2^ mg^−1^_Pt_ for Pt-CNT/GC and 13.87 cm^2^ mg^−1^_Pt_ for Pt-CNT/CNT/GC. The ECSAs determined from CO_ad_ stripping are higher than those from H_upd_ (Table 1), and the ratio of ECSA_COad_ to ECSA_Hupd_ is 1.61 for Pt-CNT/GC and 1.12 for Pt-CNT/CNT/GC. This is comparable to values reported by Mayrhofer et al. [69]. The calculated ECSAs of Pt-CNT/GC and Pt-CNT/CNT/GC are lower than the values of 30–80 cm^2^ mg^−1^_Pt_ ECSA for 2–3 nm Pt nanoparticles deposited onto CNTs as reported in the literature [70–72], in accordance with the larger size of the Pt nanoparticles. The differences in the ECSA ratios between both samples originate from the much more difficult baseline determination for the H_upd_ peaks and thus a relatively large error. In this respect, the results on the surface-specific properties (see below) are related to the ECSA determined by CO stripping, which is believed to be much more reliable due to easier baseline correction.

#### Methanol electro-oxidation

**Figure 10:**
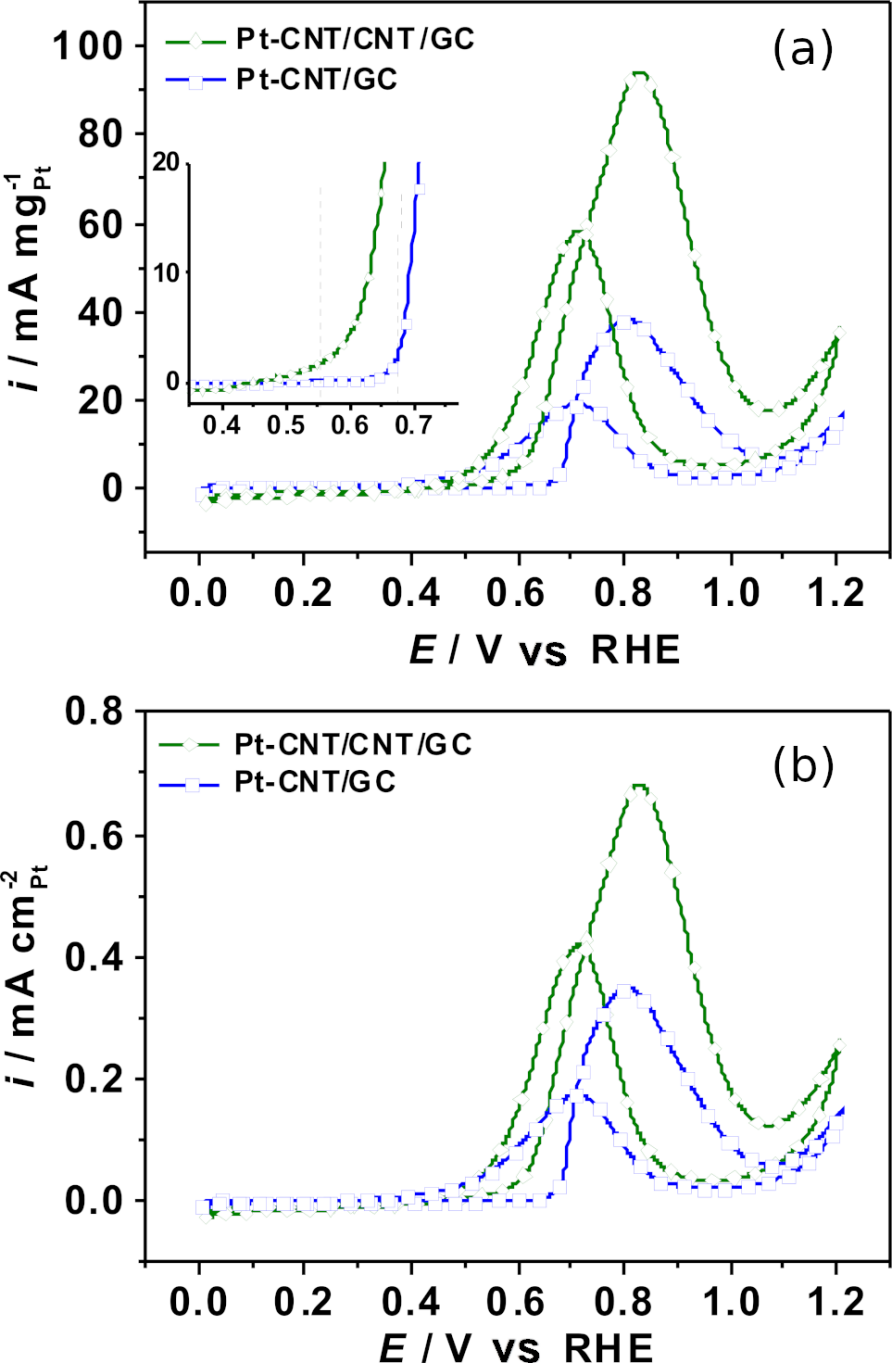
Cyclic voltammograms of Pt-CNT/GC and Pt-CNT/CNT/GC in N_2_-saturated 1 M CH_3_OH and 0.5 M H_2_SO_4_ electrolyte solution recorded at a scan rate of 5 mV s^−1^ normalized to a) Pt mass and b) Pt-ECSA evaluated by CO_ad_ stripping. The vertical dashed lines are intended as a guide for the eye.

The CVs of the methanol oxidation reaction (MOR) over the Pt-containing nanostructured electrodes were recorded in N_2_-purged 1 M CH_3_OH and 0.5 M H_2_SO_4_ aqueous solution to investigate their suitability for electrocatalytic applications. Due to the large double-layer capacity of the samples, a slow scan rate of 5 mV s^−1^ was applied. Note that the oxidation current scales with the square root of the scan rate, while the double-layer charging current linearly scales with scan rate. Thus, the slow scan rate allows for a much more reliable determination of peak potentials and currents. The fifth cycle of each measurements is represented in Figure 10. The current response for electrochemical activity towards MOR was quantified to the Pt mass and the Pt ECSA in Figure 10a and Figure 10b, respectively, where the Pt ESCA was calculated from the CO_ad_ stripping voltammograms. Figure 10 represents the typical appearance of the CVs for methanol oxidation over Pt-based catalysts. Methanol is oxidized to CO_2_ in the forward CV scan until Pt is oxidized, leading to a surface passivation and a sudden decrease in the oxidation current. During the backward CV scan, MeOH oxidation starts as soon as the electrode is liberated from oxides. In the literature, it is often observed that the current during the backward scan is higher and/or extends to less positive potentials than during the forward scan, since in the forward scan the electrode is blocked by intermediate carbonaceous species (e.g., CO) formed at lower potentials. Thus, the peak current ratio between the forward and backward scan (*i*_f_/*i*_b_) is typically used as a qualitative measure of the poisoning tolerance of a catalyst towards carbonaceous poisoning species formed during incomplete methanol oxidation at lower potentials [72–75]. In this regard, the comparably high (*i*_f_/*i*_b_) ratio (see below) indicates very good poisoning tolerance of our nanostructured samples. However, Hofstead-Duffy et al. [76] claimed that the forward and backward scan of methanol oxidation has the same chemical origin and the *i*_f_/*i*_b_ ratio is inadequate to be used as a measure for CO tolerance, which is further demonstrated and complemented in [77–78]. Thus, we attempted to obtain additional information on CO tolerance from the CO stripping voltammograms. As shown in Figure 9, the hydrogen adsorption/desorption is suppressed in the potential range from 0.05 to 0.3 V vs RHE, indicating complete coverage of Pt with CO_ad_. Pt-CNT/CNT/GC provides a more negative onset potential for CO oxidation at around 0.66 V vs RHE compared to Pt-CNT/GC (≈0.7 V). The negative shift of the onset potential indicates that Pt-CNT/CNT/GC is superior for the electro-oxidation of CO_ad_ compared to Pt-CNT/GC The reason for this improved poisoning tolerance is not known to us at the moment. However, it is known from literature that methanol as well as CO oxidation are very sensitive to Pt surface structure. It might be that a defect-rich structure of our Pt nanoparticles formed by electrodeposition is highly active for CO and MeOH oxidation and less prone to poisoning.

The cyclic voltammograms in Figure 10 show differences in terms of the electrocatalytic activity. In the forward scan, Pt-CNT/CNT/GC provides Pt mass specific and Pt surface specific peak currents of 94.46 mA mg^−1^_Pt_ and 0.68 mA cm^−2^_Pt_ at the peak potential of 0.83 V vs RHE, respectively, which is much higher than those for Pt-CNT/GC, which are 38.54 mA mg^−1^_Pt_ and 0.35 mA cm^−2^_Pt_ at 0.81 V vs RHE, respectively. Pt-GC provides much lower specific peak currents of 1.88 mA mg^−1^_Pt_ and 0.17 mA cm^−2^_Pt_ as expected. The inset in Figure 10a indicates the superior onset potential of Pt-CNT/CNT/GC (≈0.55 V vs RHE) compared to that of Pt-CNT/GC (≈0.68 V vs RHE). Pt mass specific and Pt surface specific peak current ratios of Pt-CNT/CNT/GC related to Pt-CNT/GC are 2.5 and 1.9, respectively. For the backward scans, the values for Pt-CNT/CNT/GC were 58.14 mA mg^−1^_Pt_ and 0.41 mA cm^−2^_Pt_, which are again, significantly higher than those for Pt-CNT/GC (19.74 mA mg^−1^_Pt_ and 0.17 mA cm^−2^_Pt_). These values indicate that Pt-CNT/CNT/GC provides higher catalytic activity for the methanol oxidation. Similarly, Pt-CNT/CNT/GC exhibits a 1.3 times higher surface specific current density than Pt-CNT/GC for methanol oxidation in alkaline medium as shown in Supporting Information File 1, Figure S11.

Such enhancement in specific activity could be attributed to a better distribution of Pt on the high-surface-area secondary nanotubes, while on the primary CNTs, the Pt particles may be more densely packed. Furthermore, the secondary CNTs may increase the contact between GC and primary CNTs and within the CNT network, improving electron transfer pathways. Additionally, differences in particle shape or the presence of small particles invisible to SEM may contribute, however we can only speculate on this.

In the literature, graphene/CNT hybrids were demonstrated to be superior Pt catalyst supports towards MOR with respect to graphene, CNTs or commercial carbons [45,65,79–82]. In [65] electrodeposited Pt nanoparticles were used in a similar fashion as in our paper. Using a Pt-graphene/CNT hybrid material on GC, a mass specific current of 62.02 mA mg^−1^_Pt_ was found in 1 M methanol solution at a scan rate of 50 mV s^−1^. It should be pointed that MOR measurements in literature are usually performed at scan rates of 50 or 100 mV s^−1^, while in our study, we employ 5 mV s^−1^ for reasons explained above, and increased mass-specific peak currents at higher scan rates are expected according to the Randles–Sevcik equation. Furthermore, the Pt-mass specific peak current for Pt-CNT/CNT/GC is similar to that of the Pt-graphene/CNT hybrid material on carbon cloth (101.52 mA mg^−1^_Pt_), and the Pt-surface specific peak current was two times higher than that of Pt-graphene/CNT on carbon cloth (0.34 mA cm^−2^_Pt_) reported previously [79], indicating that the introduction of secondary CNTs may provide a similar or superior beneficial effect as graphene on the electrocatalytic activity toward MOR. In general, it can be concluded that Pt-CNT/CNT/GC, as prepared in this paper, performs similar or better compared to literature studies using similar systems.

In [52], similar nanostructures were prepared that showed high activity in the oxygen reduction reaction. Although there are differences in electrode preparation (in the present case, Pt is electrodeposited onto the carbon-based electrodes, probably leading to defect-rich particles (see also below), while in [52], Pt deposition has been deposited by CVD), we think that generally the high surface area and good accessibility of the active sites is a prerequisite for the enhanced electrocatalytic performance of such structures in various electrocatalytic reactions.

## Conclusion

The preparation of hierarchically nanostructured electrodes for electrocatalytic applications was achieved via sequential growth of primary CNTs and secondary CNTs by CVD and finally Pt electrodeposition. CNT growth was carried out over electrodeposited iron nanoparticles. By varying the growth time, gas flow rate and ratio of H_2_/Ar, it was shown that the structural properties of the primary and secondary CNTs could be tuned to a certain extent. The secondary CNTs were adjusted to be smaller than the primary ones to obtain truly hierarchical structures. Enhanced double-layer capacitance as well changes in the Raman spectra with respect to the primary CNTs indicate the successful growth of secondary CNTs. Pt nanoparticles were homogeneously distributed onto both primary and secondary CNTs by electrodeposition. The Pt-CNT/CNT/GC electrode exhibited increased ECSA and electrochemical activity as well as more negative onset potential for MOR compared with Pt-CNT/GC. Additionally, CO_ad_ stripping indicated improved tolerance towards CO-like carbonaceous species poisoning. The improvement of electrochemical performance is attributed to the homogenous dispersion of Pt nanoparticles on the highly cross-linked 3D network. The prepared carbon electrode was shown to be a competitive catalyst support for methanol oxidation. In general, the applied sequences of electrodeposition and CVD steps may be considered as part of a toolbox enabling the preparation of hierarchically structured electrodes by tuning every step with respect to the requirements of a given electrochemical application.

## Experimental

### Electrode preparation

The procedure for the preparation of hierarchically structured electrodes is illustrated in Figure 1. Glassy carbon chips (GC, 2 × 1 cm^2^) were oxidized by refluxing in 5 M HNO_3_ (prepared by diluting ≥65% HNO_3_, p.a, Roth, Germany) at 100 °C for 2 h to activate their surface and form oxygen functional groups as anchoring sites. Afterwards, Fe nanoparticles were grown on the oxidized GC by double pulse deposition [57] in 0.005 M FeSO_4_·7H_2_O (≥99.5%, Roth, Germany) and 0.5 M MgSO_4_·7H_2_O (pure, Roth, Germany) aqueous solution. MgSO_4_ simply serves as a conducting electrolyte to avoid high solution resistance and does not take part in the reaction. A potential sequence consisting of a so-called “no-effect potential” (*E* = −0.75 V vs Ag|AgCl|KCl_sat._; *t* = 5 s), a nucleation potential (*E* = −1.41 V vs Ag|AgCl|KCl_sat._; *t* = 0.2 s) and a growth potential (*E* = −1.27 V vs Ag|AgCl|KCl_sat._; *t* = 12 s) was applied. The potentials were estimated considering linear-sweep voltammograms recorded in the potential range between −0.5 V and −1.75 V vs Ag|AgCl|KCl_sat._ with a scan rate of 5 mV s^−1^ as shown in Supporting Information File 1, Figure S1. The deposited Fe nanoparticles serve as a catalyst for growth of the so-called “primary CNTs”, which was carried out through CVD at 750 °C in H_2_/Ar mixtures saturated with cyclohexane (Roth) at room temperature, and the resulting structures are labelled as CNT/GC. The influence of growth time (30 min, 60 min and 120 min), gas flow rate and H_2_/Ar ratio on the CNT growth was investigated, where gas flow rates were adjusted by mass flow controllers (Bronkhorst High-Tech, Germany). Prior to CVD, the Fe catalysts were conditioned at 750 °C for 30 min in a H_2_/Ar gas mixture. After the CNT growth, the surface of CNT/GC is highly hydrophobic. To remove remaining Fe nanoparticles, the CNT/GC electrodes were immersed in concentrated HNO_3_ at room temperature for 12 h, where the CNTs were also oxidized to form anchoring sites for a second Fe deposition, which was carried out in the same way as above but with 8 s of growth time. “Secondary CNTs” were grown on the Fe-CNT/GC material in a gas mixture of H_2_ (1.2 L h^−1^) and Ar (0.5 L h^−1^) at 750 °C for 120 min to form the hierarchical CNT/CNT/GC structure. Afterwards, the Fe nanoparticles were again leached out in concentrated HNO_3_. Furthermore, the same procedure was performed using acetonitrile as a carbon source to yield N-CNT/N-CNT/GC.

Finally, Pt nanoparticles were electrochemically deposited onto CNT/GC and CNT/CNT/GC in an aqueous 0.005 M Pt(NO_3_)_2_ and 0.1 M NaNO_3_ solution via linear-sweep voltammetry from 0 to −0.9 V vs Ag|AgCl|KCl_sat_ at a scan rate of 5 mV s^−1^ to form Pt-CNT/GC and Pt-CNT/CNT/GC. For comparison, Pt deposition onto GC was performed in the same manner. The amount of deposited Pt was calculated from the charge consumed during the linear sweep voltammetry according to the following faradic reaction (Equation 1) and Faraday’s law (Equation 2):

[1] $${\text{Pt}}^{2+}+2e^{-}\to \text{Pt}$$

[2] $$Q_{\text{Pt}}=n\times \frac{m_{\text{Pt}}}{M_{\text{Pt}}}\times F$$

where *Q*_Pt_ is the charge consumed to reduce Pt ions to Pt, *n* is the number of transfer electrons, *m*_Pt_ is the amount of Pt, *M*_Pt_ is the atomic weight of Pt (195.09 g mol^−1^), and *F* is Faraday’s constant (96485.31 C mol^−1^).

### Electrochemical characterization

Electrochemical experiments were carried out at room temperature in a one-compartment three-electrode cell employing a Gamry potentiostat PGI 4 controlled by the Gamry Framework 2.67 software. The modified GC, after its various treatment steps, served as working electrode, a Pt mesh (GoodFellow, Germany) as the counter electrode, and an Ag|AgCl|KCl_sat._ (SE20, Sensortechnik Meinsberg, Germany) for the electrodeposition and methanol oxidation or a reversible hydrogen electrode (RHE) built in-house for characterization as reference electrode. Before Pt deposition, GC, oxidized GC, CNT/GC and CNT/CNT/GC, were cleaned and activated employing CV in the potential range between 0 V and 1.2 V vs RHE at a scan rate of 200 mV s^−1^ for 50–100 cycles in N_2_-purged 0.5 M H_2_SO_4_ (prepared from 98% H_2_SO_4_, Roth, Germany) aqueous solution until the CVs did not change any more, while Pt-GC, Pt-CNT/GC and Pt-CNT/CNT/GC were cycled in the potential range from 0.05 V to 1.2 V vs RHE. After this treatment, the double-layer current of the electrodes without Pt and the hydrogen adsorption/desorption (H_ads/des_) of the electrodes containing Pt were determined by CV at 100 mV s^−1^ in a fresh N_2_-purged aqueous 0.5 M H_2_SO_4_ solution. The average charge during H_ads_ and H_des_ was used to determine the Pt-electrochemical surface area (ECSA). Additionally, the ECSA was determined through CO_ad_ stripping voltammetry measured at a scan rate of 20 mV s^−1^ in the potential range of 0.05–1.1 V vs RHE in 0.1 M HClO_4_ solution. HClO_4_ was used as a supporting electrolyte in this case to avoid changes/deviations in the CO stripping peak by sulfate/bisulfate adsorption. The solution was purged with CO for 20 min to allow for CO adsorption on the Pt catalyst, and excess CO was removed by purging the electrolyte with N_2_ for 20 min. The working electrode was held at 0.05 V during this procedure until the stripping voltammogram was recorded. Afterwards, the activity of the Pt-containing electrodes for methanol oxidation was investigated by CV at a low scan rate of 5 mV s^−1^ in an N_2_-purged 1 M CH_3_OH and 0.5 M H_2_SO_4_ aqueous solution. The low scan rate was used because of the large double-layer capacity of the hierarchical electrodes.

### Structural characterization

The nanostructured electrodes were examined via SEM employing an ESEM XI 30 FEG (Philips, Germany) instrument to characterize the morphology and structural properties. The average particle size and size distribution of Fe nanoparticles were determined by examining the size of 200–300 particles with the software “Lince” (TU Damstadt, Germany) [83]. Raman spectra were measured employing a Renishaw InVia spectrometer with 532 nm excitation wavelength from a Cobolt CW DPSS laser. Due to the considerably thin film of the CNT layers, and thus the low amount of Pt, XRD did not yield useful results regarding the Pt structure and particle size.

## Supporting Information

File 1 Additional SEM images and results of electrochemical characterization.
