# Supplementary material for: Hierarchically structured 3D carbon nanotube electrodes for electrocatalytic applications
Source: Beilstein J Nanotechnol. 2019 Jul 24;10:1475–87. doi: 10.3762/bjnano.10.146 (PMC6664386; doi:10.3762/bjnano.10.146)
Supplement: File 1 — Additional SEM images and results of electrochemical characterization. [file Beilstein_J_Nanotechnol-10-1475-s001.pdf]

BEILSTEIN JOURNAL OF NANOTECHNOLOGY

Supporting Information

for

Hierarchically structured 3D carbon nanotube electrodes for electrocatalytic applications

Pei Wang, Katarzyna Kulp and Michael Bron

Beilstein J. Nanotechnol. **2019,** *10,* 1475–1487. doi:10.3762/bjnano.10.146

Additional SEM images and results of electrochemical characterization

License and Terms: This is a supporting information file under the terms of the Creative Commons Attribution License (http://creativecommons.org/licenses/by/4.0). Please note that the reuse, redistribution and reproduction in particular requires that the authors and source are credited.
The license is subject to the *Beilstein Journal of Nanotechnology* terms and conditions: (https://www.beilstein-journals.org/bjnano)

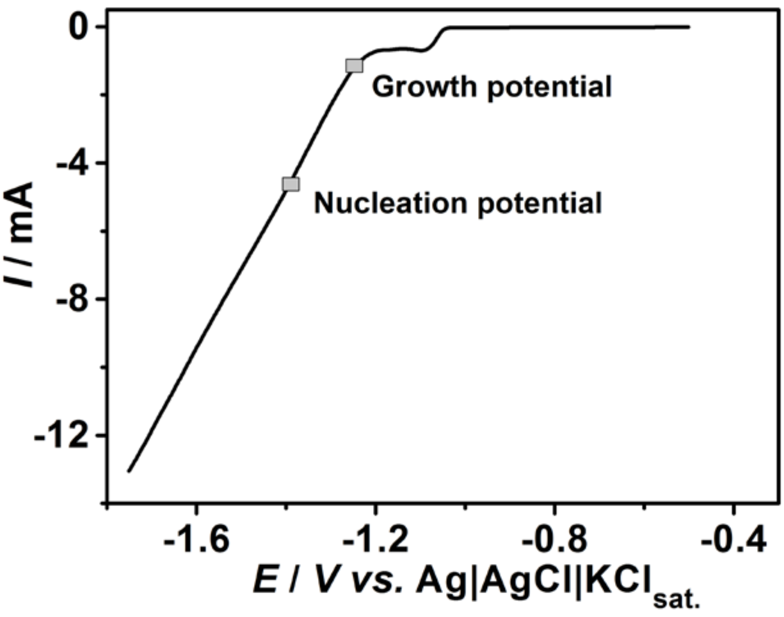

Figure S1: Single sweep voltammogram of iron deposition on oxidized GC recorded in the potential range between -0.5 V and -1.75 V vs Ag|AgCl|KCl$_{sat.}$ with a scan rate 5 mV s^{-1} in N_2 purged 0.005 M $FeSO_4$ * 7 H_2O and 0.5 M $MgSO_4$ * 7 H_2O aqueous solution. The respective potentials for double-pulse deposition are indicated.

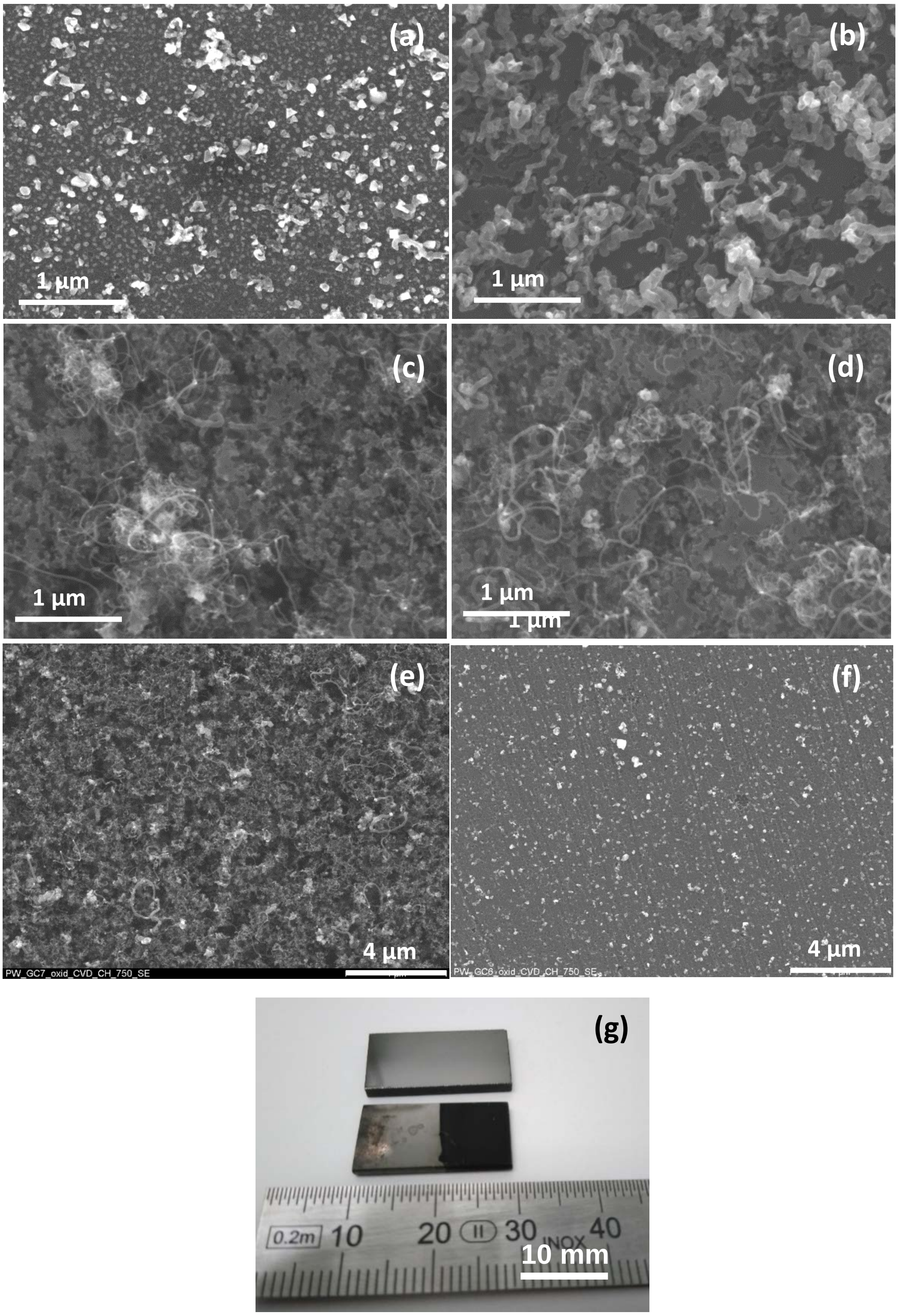

Figure S2: SEM images of CNTs grown at 750 °C using cyclohexane as carbon source through CVD with an H_2/Ar ratio of 1.8: (a) for 30 min and (b) for 60 min using 1.7 L h^{-1} as total gas flow rate; (c) with 6.7 L h^{-1} as total gas flow rate and (d) 12.1 L h^{-1} as flow rate for 120 min; as well as with a gas flow rate of 1.7 L h^{-1} for 120 min with different H_2/Ar ratios: (e) 1.4 (1.0 L h^{-1}/0.7 L h^{-1}), (f) 2.4 (1.2 L h^{-1}/0.5 L h^{-1}), (g) optical images of GC before and after CNTs growth for 120 min.

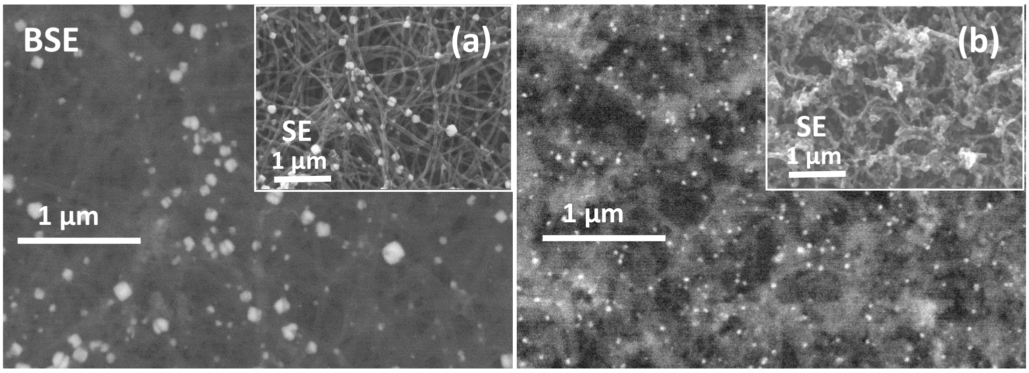

Figure S3: SEM and BSE images of Fe nanoparticles deposited onto primary CNTs on GC via the above mentioned electrochemical deposition with different deposition times: (a) 12 s and (b) 6 s.

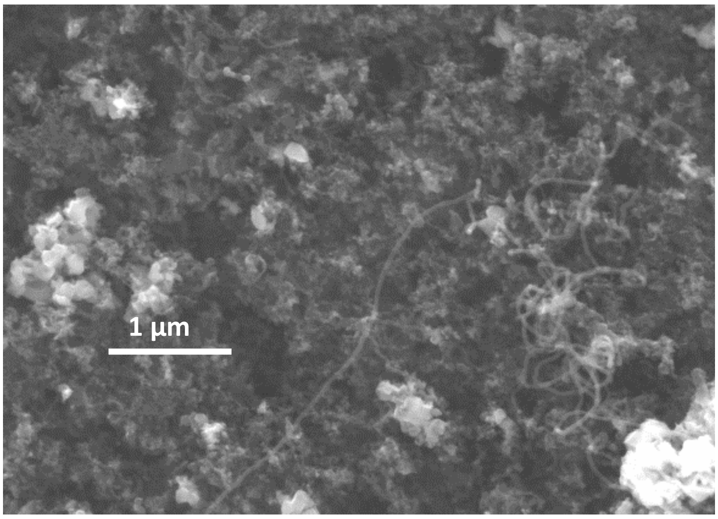

Figure S4: SEM images of secondary CNT growth at 750 °C for 120 min with optimized H_2/Ar ratio of 1.8 (1.1 L h^{-1}/ 0.6 L h^{-1}).

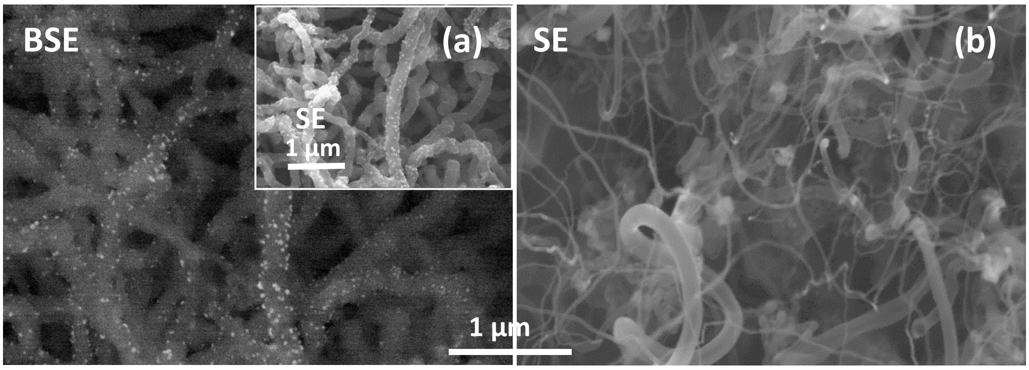

Figure S5: SEM and BSE images of (a) electrodeposited secondary Fe nanoparticles onto thicker CNTs and GC with 6 s deposition time as well as (b) secondary CNTs grown via optimized growth condition. (The CNTs with increased diameter were grown at bigger primary Fe nanoparticles created by longer deposition time.)

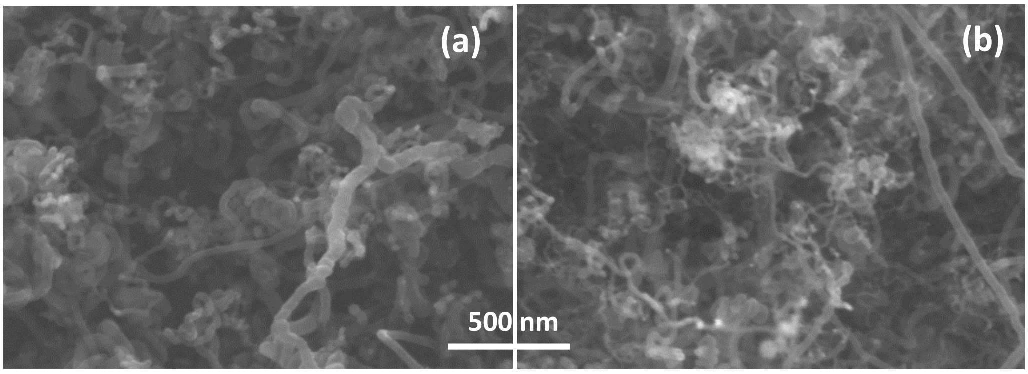

Figure S6: SEM images of CNTs before and after secondary CNT growth with acetonitrile as carbon source at 750 °C for 120 min.

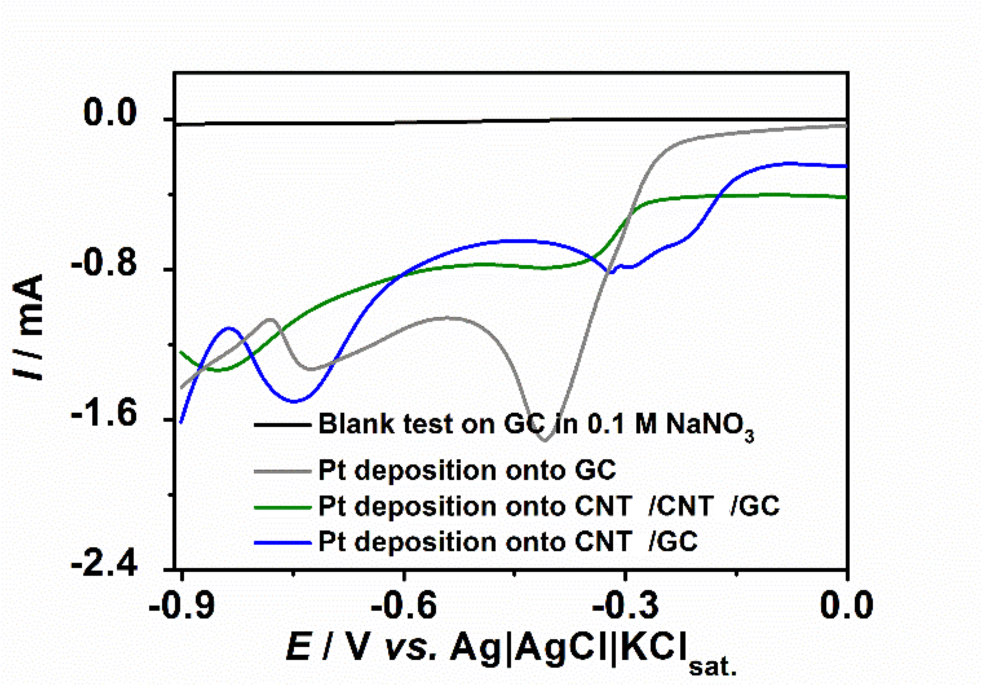

Figure S7: Linear sweep voltammogram for Pt deposition recorded at a scan rate of 5 mV s^{-1} from 0 to -0.9 V vs Ag|AgCl|$KCl_{sat.}$ in a 0.005 M $Pt(NO_3)_2$ and 0.1 M $NaNO_3$ solution.

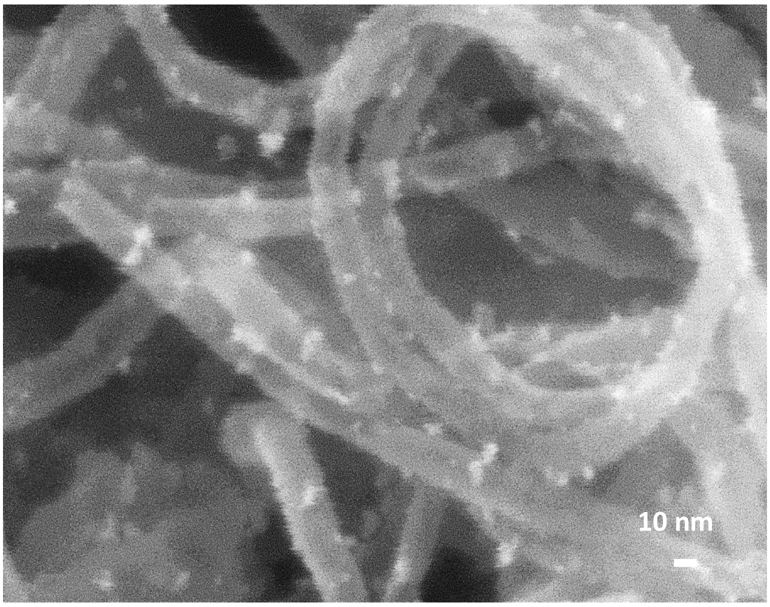

Figure S8: SEM images of Pt nanoparticles deposited onto CNT/CNT/Pt from a 0.005 M $Pt(NO_3)_2$ and 0.1 M $NaNO_3$ aqueous solution via linear-sweep voltammetry from 0 to -0.9 V vs. Ag|AgCl|$KCl_{sat.}$ at 5 mV s^{-1} scan rate.

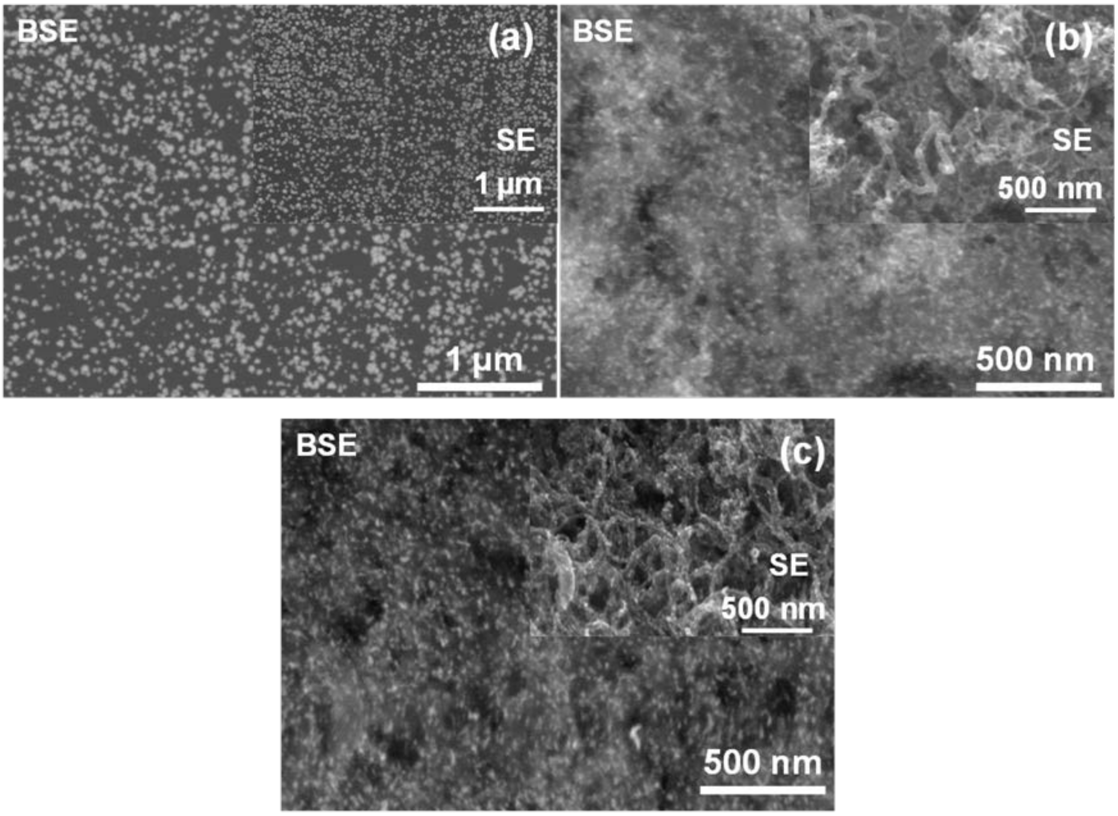

Figure S9: SEM und BSE images of Pt nanoparticles deposited from an aqueous 0.005 M $Pt(NO_3)_2$ and 0.1 M $NaNO_3$ solution via single-sweep voltammetry from 0 to -0.9 V vs. Ag|AgCl|$KCl_{sat.}$ at a scan rate of 5 mV s^{-1} onto: (a) oxidized GC, (b) CNT/GC and (c) CNT/CNT/GC.

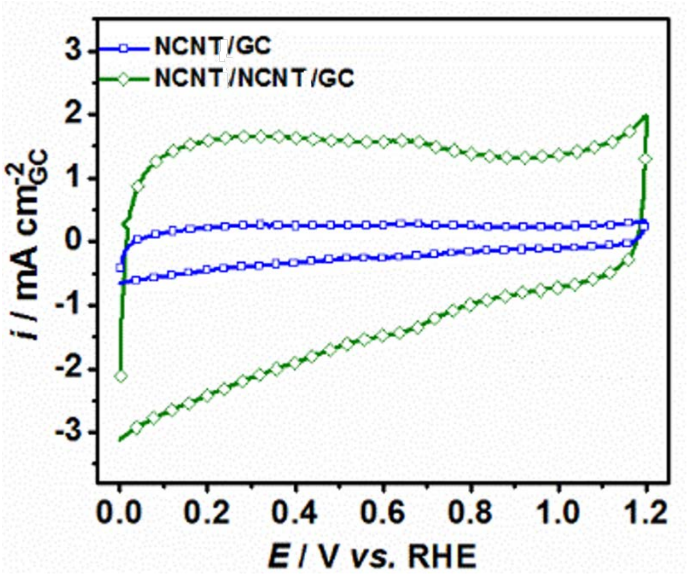

Figure S10: Cyclic voltammograms of NCNT/GC and NCTN/NCNT/GC electrodes recorded at a 100 mV s^{-1} scan rate in 0.5 M H_2SO_4 aqueous solution purged with N_2 at room temperature.

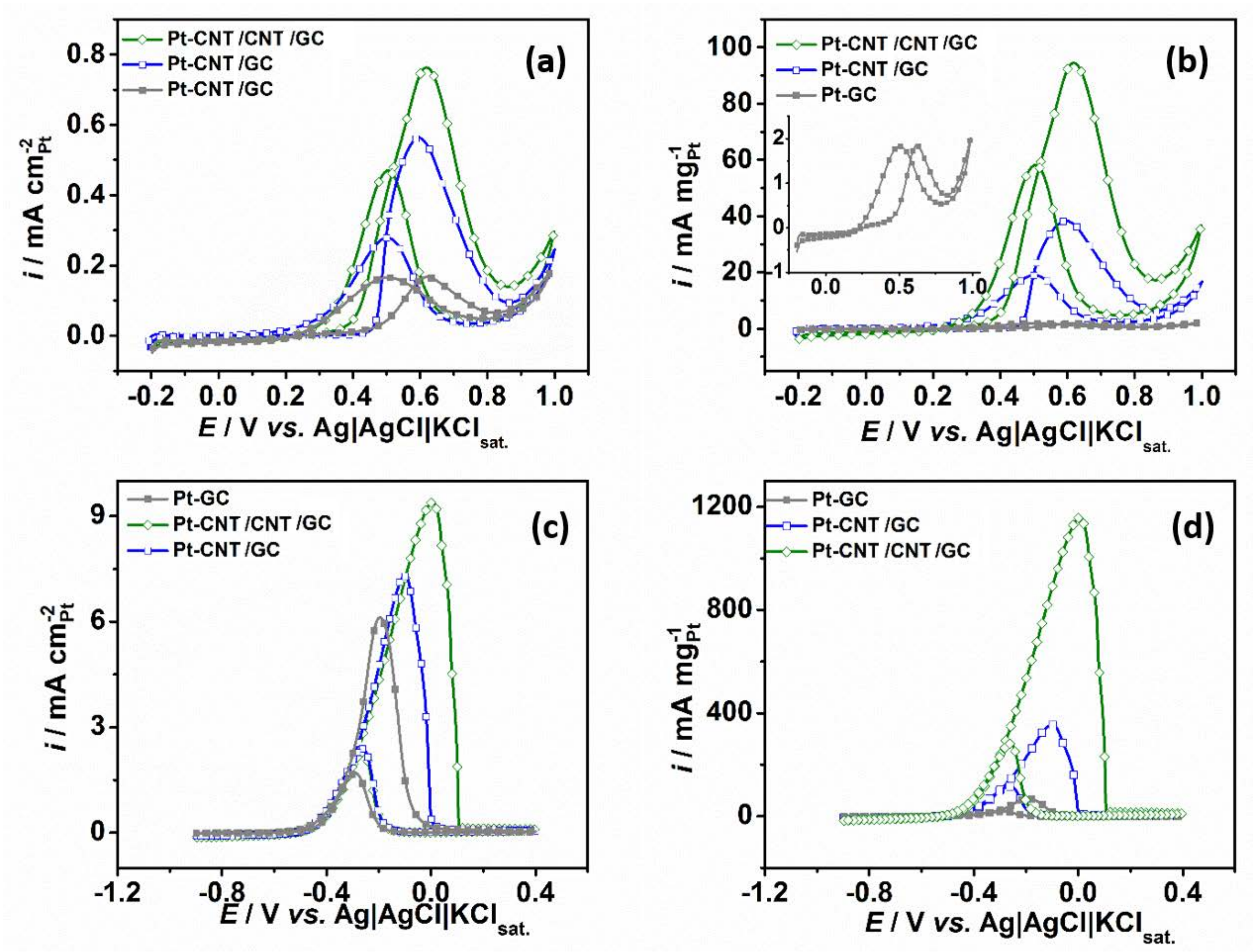

Figure S11: Cyclic voltammograms of Pt-GC, Pt-CNT/GC and Pt-CNT/CNT/GC recorded at a scan rate of 5 mV s^{-1} respectively normalized to Pt-ECSA evaluated by $H_{ads/des}$ and Pt mass in the potential range from -0.2 V–1.0 V vs. Ag|AgCl|KCl_{sat} in N_2 purged 1 M CH_3OH and 0.5 M H_2SO_4 solution (a,b) as well as in potential range of -0.9 V–0.4 V in 1 M CH_3OH and 0.5 M KOH solution (c,d).
